# Recipes for Determining Doneness in Poultry Do Not Provide Appropriate Information Based on US Government Guidelines

**DOI:** 10.3390/foods7080126

**Published:** 2018-08-09

**Authors:** Edgar Chambers, Sandria Godwin, Taylor Terry

**Affiliations:** 1 Center for Sensory Analysis and Consumer Behavior, Kansas State University, 1310 Research Park Dr., Manhattan, KS 66502, USA; tlterry@ksu.edu; 2 Department of Human Sciences, College of Agriculture, Tennessee State University, 3500 John A. Merritt Boulevard, Nashville, TN 37209, USA; sgodwin@tnstate.edu

**Keywords:** food safety, poultry, consumer, cooking, behavior

## Abstract

Research has shown that consumers use unsafe food handling practices when preparing poultry, which can increase the risk of foodborne illness such as salmonellosis or campylobacteriosis. Recipes from cookbooks, magazines, and the internet commonly are used as sources for consumers to prepare food in homes and the expectation is that food will be safe when prepared. According to the United States Department of Agriculture (USDA), using a thermometer properly is the only way to accurately check for doneness of poultry. The objective of this study was to assess poultry recipes, including recipes for whole birds and poultry parts, to determine if food safety information concerning thermometer use was included within the recipe. Poultry recipes (*n* = 474) were collected from 217 cookbooks, 28 magazines, 59 websites, and seven blogs. Approximately 33.5% of the recipes contained a specific temperature for doneness, with 73% of those cooked to ≥165 °F/74 °C, as recommended by USDA. Ninety-four percent of recipes used cooking time and about half of the recipes used visual measurements, such as color or juices running clear, to determine doneness. This study showed that most recipes do not contain appropriate information to assure safe cooking of poultry by consumers. Modifying recipes by adding food safety information, such as thermometer use and proper temperatures, could increase the use of proper food preparation behaviors by consumers.

## 1. Introduction

Foodborne illness, including that from poultry products, continues to be a major public health burden, resulting in illness and mortality at national and global levels [1]. In the United States alone, 48 million people are affected by foodborne illness annually [2]. A common cause of foodborne illnesses is poultry, such as chicken, that can result in incidences of salmonellosis and campylobacteriosis, which, when combined, account for 1.8 million cases of foodborne infections in the United States [2]. This makes focusing on poultry food safety pertinent to efficiently decreasing foodborne illness.

Many foodborne infections in the United States occur from improper food handling practices in the home [3]. According to Healthy People 2020, cooking foods to a safe internal temperature is an area needing the most improvement among consumers [4]. Temperature plays an important role in the safety of foods when consumed, especially in poultry. The United States Department of Agriculture (USDA) recommends that all poultry, whole, pieces, and ground, be cooked to a minimum internal temperature of 165 °F/74 °C [5]. Consumers are aware of the risk of foodborne illness from undercooked foods, but continue not to use thermometers to determine doneness [6,7,8,9]. Hicks [10] states that consumers have a responsibility to handle and prepare food properly. Unfortunately, consumers still use subjective measures, such as appearance, to determine the doneness of foods, which is an unsafe practice [7,8,11,12]. Maughan [13] discussed that color of products can change depending on lighting conditions.

Research has shown the need to improve food-handling practices among consumers who cook poultry [12]. Consumers obtain food safety information from many sources such as cookbooks, government publications, food labels, television, and health professionals [14,15,16,17]. Although cookbooks and recipes can be sources for cooking information for consumers, food safety information is not prevalent within most recipes [18,19,20,21]. In addition to cooking on top of the stove or in the oven, outdoor grilling or ‘barbecuing’, is a tradition for many U.S. consumers, especially in warm months. Chicken is one of the most common foods that consumers prepare on the grill [22]. In addition, more consumers are starting to grill year-round. 

Research concerning food safety information of recipes for cooking poultry, including grilling, is lacking. Thus, the objective of this study was to evaluate poultry recipes based on the inclusion of temperature information, determination of doneness methods, and other food safety information.

## 2. Materials and Methods 

Recipes that called for cooking poultry, either chicken or turkey, whole or in parts, were analyzed. Recipes from multiple sources, both online (internet recipe sites and blogs) and in print (cookbooks and magazines) were selected for analysis. Sources in print were found at the university library, local public library, or cookbooks provided by friends and family. Online sources were found by searching “poultry (or chicken or turkey) recipes” in the Google search engine. No source was older than the year 2000 in order to ensure reasonably current information. To qualify, the recipe had to be obtained from either a primary ‘cookbook’ (books about dieting or lifestyle that included a few recipes were not selected), magazines where recipes are a typical section or category, or other sources that publish recipes as a primary part of their presence (any of those categories could be in-print or online). 

### Recipe Selection and Analysis

Because of the number of sources available, in most cases only one recipe and in no cases were more than three recipes selected from any single source. Only in the case of large online resources or extensive cookbooks focused on poultry was more than one-recipe selected. An attempt was made to select many different types of sources and many types of recipes among those available, e.g., baking, frying, grilling, stewing, etc. 

For print sources, if a large number of poultry recipes were given in the source, the number of poultry recipes were counted from the index. A random number generator (random.org) was used to generate up to three numbers from one to the number of recipes that were in the source. The numbers generated were used to select the recipes in the order of which they were presented in the source. For example, if there were fifteen poultry recipes in a cookbook, the number generator would produce one number from one to fifteen. If the number generated was nine, then the ninth recipe was selected for analysis. For online recipes or if an index was not available, the first one to three poultry recipes that were viewed within the source were selected. 

**Figure 1:**
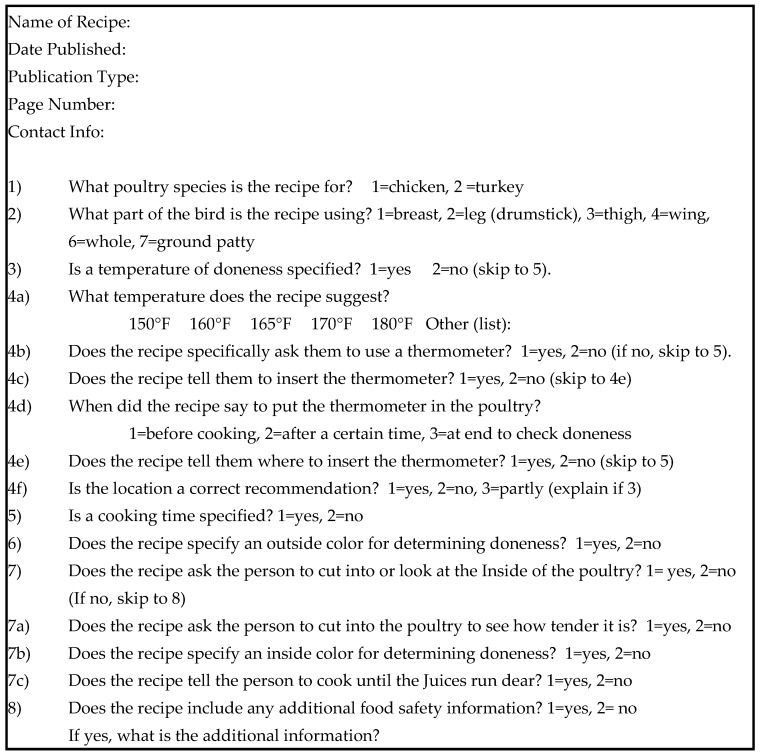
Checklist for evaluating recipe information related to doneness.

A checklist (Figure 1) was created to evaluate all recipes uniformly. Recipes were analyzed based on temperature information and determinants of doneness of the poultry specified within the recipe. Recipes also were assessed to determine correct thermometer usage. Although any additional food safety information included in the recipe also was noted, rarely was any included and, thus, is not summarized in this paper. 

## 3. Results and Discussion

In total, 474 recipes were analyzed from 311 sources. There were 410 chicken recipes and 64 turkey recipes. Recipes were collected from 217 cookbooks, 28 magazines, 59 websites, and 7 blogs. 

### 3.1. Endpoint Temperatures

**Figure 2:**
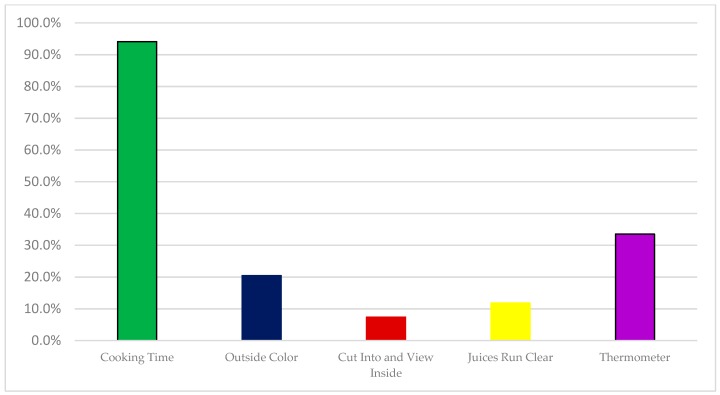
Methods suggested in recipes for determining doneness of poultry (*n* = 474 recipes).

Of the 474 recipes analyzed, only 33.7% of the recipes recommended use of a thermometer to determine doneness (Figure 2). Most recipes simply stated a cooking time and 95% of recipes provided a cooking time even when other methods of detecting doneness were suggested. Maughan et al. [8] showed that when a thermometer was not used, almost 30% of cooks undercooked either a chicken breast or ground turkey patties. This suggests that simply using cooking time may be problematic. Using time assumes that the heating pattern of the appliances used by the consumer matches that used in the recipe development—correct oven or oil temperature, consistent heat source, and similar placement on a grill, for example. Unfortunately, two recent studies [11,23] in the U.S. and Canada both show low rates of thermometer use (11–26%) when cooking poultry parts (such as legs or breasts) or ground poultry patties. In addition, it is well known that examining juice color is not accurate in judging doneness as suggested by recipes that include suggestions such as “until juice runs clear”. Hoey [24] stated that chicken meat is no longer pink and the “juices run clear” at 180 °F, quite a bit higher than the recommended safe cooking temperature. 

**Figure 3:**
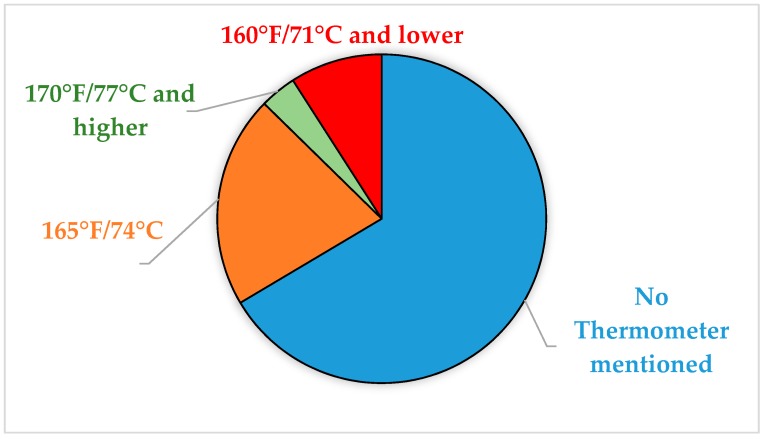
Temperature information provided by recipes in this study (%).

Recipes that specified using a thermometer provided specific endpoint temperature within the recipe. Almost 74% of those that specified using a thermometer provided a correct temperature of 165 °F/74 °C (Figure 3). Approximately 9.1% recommended a temperature too low and 2.6% recommended a temperature greater than 165 °F/74 °C. The recipes that recommend too low a temperature is putting consumers at risk of foodborne illness while the ones recommending too high a temperature risk overcooking and providing poor sensory quality. 

### 3.2. Thermometer Placement Information

Even though some recipes provided temperature information and many of those provided a correct temperature, only 30.1% (*n* = 49) of the recipes that specified a temperature provided information on the correct location to insert the thermometer. Of those recipes that did provide thermometer placement information, approximately half gave either incorrect or partly incorrect information for location. If the thermometer is inserted into the wrong location, the temperature readings will be inaccurate [25]. Maughan and other [8] found that 36% of consumers who used a thermometer, used it incorrectly. This study suggests that recipes could do more to provide information on thermometer insertion as well. 

### 3.3. General Findings

Of course, of greatest concern are the recipes that provide no temperature information. Many of the recipes relied on time and/or subjective measurements, such as appearance and color, to determine the doneness of the poultry rather than using a thermometer. Using visual indicators to determine the doneness of poultry is an unsafe practice as it can result in undercooked poultry. Two studies [8,26] found that visual inspections of doneness by consumers were inaccurate producing some samples that were undercooked, including some that contained active Campylobacter *jejuni* cells [26]. 

The analysis of the recipes showed that consumers are not receiving the proper food safety information within recipes for preparing poultry. Although many of the cookbooks contained some food safety information, such as endpoint temperatures of poultry and other meats in the introduction or appendix of the book, it is not guaranteed that consumers will be aware of that information if they are not reading the whole book. Understanding and using easily adoptable strategies that maintain high levels of food safety and food quality are important [27]. Therefore, food safety information could be added within the recipes to alter food safety behaviors of consumers [20,22]. Maughan and others [14] showed that simple modifications of recipes to include specific food safety steps, such as use of thermometers, dramatically increased safe food preparation practices. Based on this and other studies, we suggest that modification of recipes with the addition of food safety information, such as handwashing, sanitation to reduce cross-contamination, and use of thermometers with proper temperature and placement information would improve food-handling practices and increase thermometer use among consumers. 

## 4. Conclusions

Research examining recipe information related to doneness when cooking poultry shows that there is considerable variability of information and generally does not provide recommended information such as use and placement of thermometers. Such information could easily be added to recipes and should be considered by publications promoting consumer cooking using recipes. With little expense or change in their recipes, publishers (both print and online) as well as popular bloggers could help to increase awareness of safe cooking of poultry by providing appropriate food safety and sanitation steps as well as providing doneness directions in recipes though use of thermometers, accurate placement of thermometers, and correct end-point temperatures.
